# Complete Genome Sequence of a Legionella longbeachae Serogroup 2 Isolate Derived from a Patient with Legionnaires’ Disease

**DOI:** 10.1128/MRA.01563-19

**Published:** 2020-01-30

**Authors:** Jan Haviernik, Krista Dawson, Trevor Anderson, David Murdoch, Stephen Chambers, Patrick Biggs, Simone Cree, Sandy Slow

**Affiliations:** a Department of Virology, Veterinary Research Institute, Brno, Czech Republic; b Faculty of Science, Masaryk University, Brno, Czech Republic; c Department of Pathology and Biomedical Science, University of Otago, Christchurch, New Zealand; d Microbiology, Canterbury Health Laboratories, Christchurch, New Zealand; e Molecular Epidemiology and Veterinary Public Health Laboratory, Infectious Disease Research Centre, School of Veterinary Science, Massey University, Palmerston North, New Zealand; Loyola University Chicago

## Abstract

Legionella longbeachae is the predominant cause of Legionnaires’ disease (LD) in New Zealand. Although serogroup 2 (sg2) does not contain the most clinically significant strain, it is an important cause of disease. Here, we report the complete genome sequence of an sg2 isolate from a patient who was hospitalized with LD.

## ANNOUNCEMENT

Bacteria of the genus Legionella are ubiquitous in both natural and human-made environments, where they are intracellular parasites of free-living amoebae (1, 2). Humans become accidental hosts following exposure to contaminated materials; infection of lung macrophages can lead to Legionnaires’ disease (LD), a type of pneumonia that can be fatal. Of the more than 20 different species that have been reported to cause human disease, Legionella longbeachae is the most important in New Zealand, causing nearly two-thirds of all notified cases (3–7). Although L. longbeachae serogroup 1 (sg1) is the most clinically significant, a small number of LD cases are caused by serogroup 2 (sg2) strains. Currently, there are only two draft genome sequences available for *L. longbeachae* sg2 (strains 98072 and C-4E7) (8). Here, we report the complete genome sequence of a *L. longbeachae* sg2 isolate obtained from a bronchial wash sample from a patient who was hospitalized with LD in 2008 in Christchurch, New Zealand.

The isolate was grown on buffered charcoal-yeast extract agar (72 h, 35°C), and DNA was purified using the GenElute bacterial genomic DNA kit (Sigma-Aldrich, MO, USA). Sequencing was conducted using the GridION Nanopore (Oxford Nanopore Technologies [ONT], UK) and Illumina MiSeq (San Diego, CA) systems. For GridION sequencing, the DNA was further concentrated using pellet paint coprecipitant (Novagen, Merck, Germany) to obtain at least 50 ng/μl, which was quantified by Qubit fluorometry (Thermo Fisher Scientific, Waltham, MA). The library was prepared for GridION Nanopore sequencing following the protocol of the rapid barcoding sequencing kit (catalog no. SQK-RBK002 [ONT]), in which 400 ng of genomic DNA underwent tagmentation followed by sequence adaptor ligation, with DNA purification between each step. The library was sequenced on the GridION system for 24 h with high-accuracy base calling with the R9.4.1 flow cell, and the reads were demultiplexed using Guppy barcoding software version 3.1.5+ (ONT). The MiSeq library was prepared using the Nextera XT library prep kit and sequenced using the MiSeq reagent kit v2 (500 cycles). The total yield for one-directional high-quality GridION reads was 112,806, while 890,000 MiSeq paired-end reads were obtained. The short and long reads were assembled using Flye software version 2.4.2 (9) and Unicycler software version 4.7 (10) with default parameters.

A single closed genome was constructed, consisting of a 4,199,426-bp chromosome (GC content, 37.1%; 75× coverage) and a 150,432-bp plasmid (GC content, 38.1%; 89× coverage), which is larger than the other sg2 draft genomes (C-4E7 and 98072 had a chromosome of 3,979,000 bp and 4,018,000 bp, respectively, and each strain contained a 133,800-bp plasmid [8]). Annotation was performed using the NCBI Prokaryotic Genome Annotation Pipeline (PGAP) version 4.8 (11), which predicted 3,735 coding sequences, 12 rRNAs, 48 tRNAs, and 5 noncoding RNAs (ncRNAs). Alignment using Mauve version 2015-02-13 (12) revealed that our sg2 chromosome was 122.1 kb, 36.7 kb, and 56.5 kb larger than the chromosome of *L. longbeachae* sg1 isolates NSW150 (GenBank accession number NC_013861), FDAARGOS_201 (accession number NZ_CP020412), and F1157CHC (accession number NZ_CP020894) (13), respectively, while the sg2 plasmid was 78.6 kb and 42.2 kb larger than the NSW150 and F1157CHC plasmids, respectively.

### Data availability.

The GridION and Illumina MiSeq sequence reads described here have been deposited at NCBI/GenBank under BioProject number PRJNA557074. The whole-genome sequence described here has been deposited at NCBI/GenBank under accession numbers CP042254 (chromosome) and CP042253 (plasmid).
